# Changing the tracks: screening for electron transfer proteins to support hydrogen production

**DOI:** 10.1007/s00775-022-01956-1

**Published:** 2022-08-29

**Authors:** Alexander Günzel, Vera Engelbrecht, Thomas Happe

**Affiliations:** Faculty of Biology and Biotechnology, Photobiotechnology, Ruhr-University Bochum, Universitätsstraße 150, 44801 Bochum, Germany

**Keywords:** Ferredoxin, [Fe–Fe]-hydrogenase, FNR, Iron–sulfur clusters, De novo peptide design, *Chlamydomonas reinhardtii*

## Abstract

**Abstract:**

Ferredoxins are essential electron transferring proteins in organisms. Twelve plant-type ferredoxins in the green alga *Chlamydomonas reinhardtii* determine the fate of electrons, generated in multiple metabolic processes. The two hydrogenases HydA1 and HydA2 of. *C. reinhardtii* compete for electrons from the photosynthetic ferredoxin PetF, which is the first stromal mediator of the high-energy electrons derived from the absorption of light energy at the photosystems. While being involved in many chloroplast-located metabolic pathways, PetF shows the highest affinity for ferredoxin-NADP^+^ oxidoreductase (FNR), not for the hydrogenases. Aiming to identify other potential electron donors for the hydrogenases, we screened as yet uncharacterized ferredoxins Fdx7, 8, 10 and 11 for their capability to reduce the hydrogenases. Comparing the performance of the Fdx in presence and absence of competitor FNR, we show that Fdx7 has a higher affinity for HydA1 than for FNR. Additionally, we show that synthetic FeS-cluster-binding maquettes, which can be reduced by NADPH alone, can also be used to reduce the hydrogenases. Our findings pave the way for the creation of tailored electron donors to redirect electrons to enzymes of interest.

**Graphical abstract:**

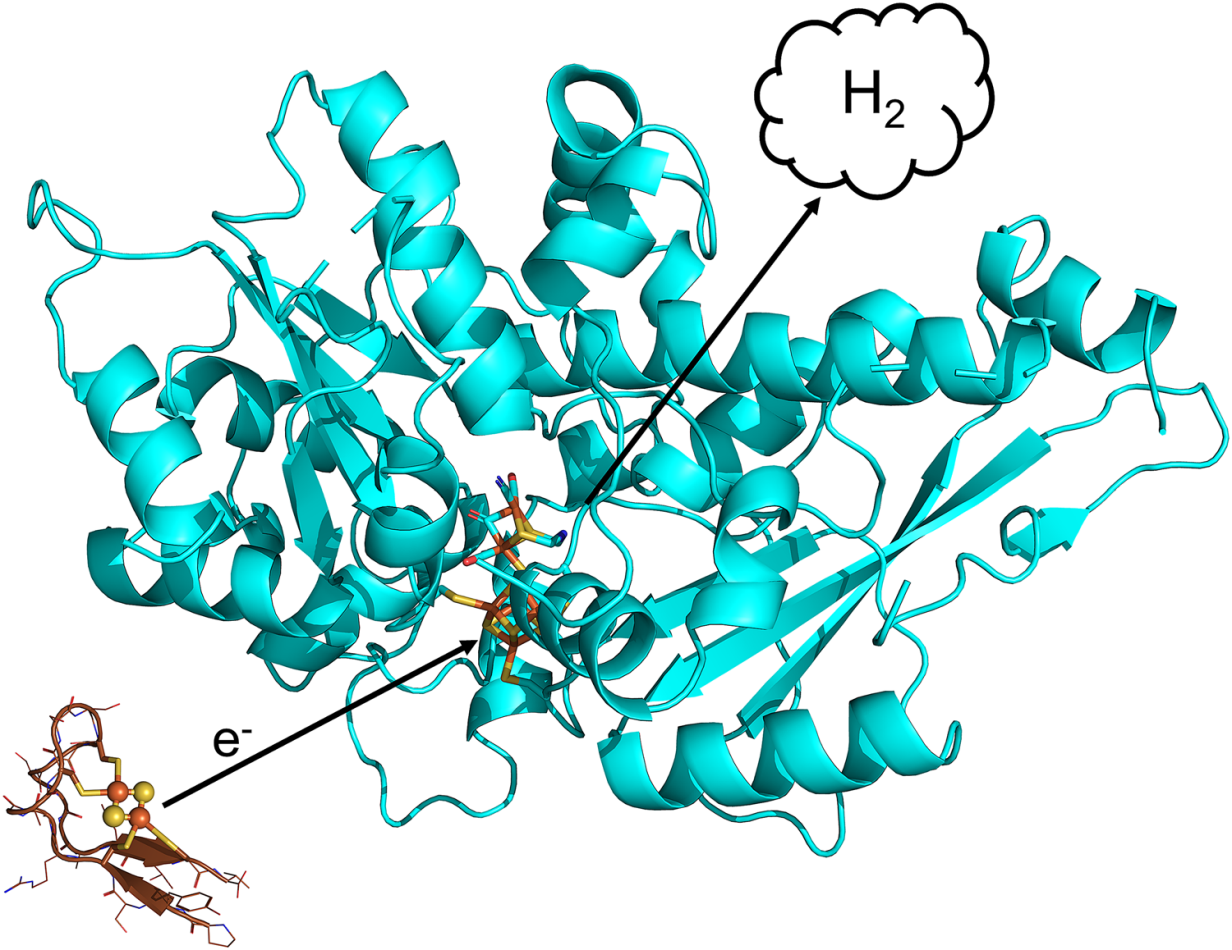

**Supplementary Information:**

The online version contains supplementary material available at 10.1007/s00775-022-01956-1.

## Introduction

**Fig. 1:**
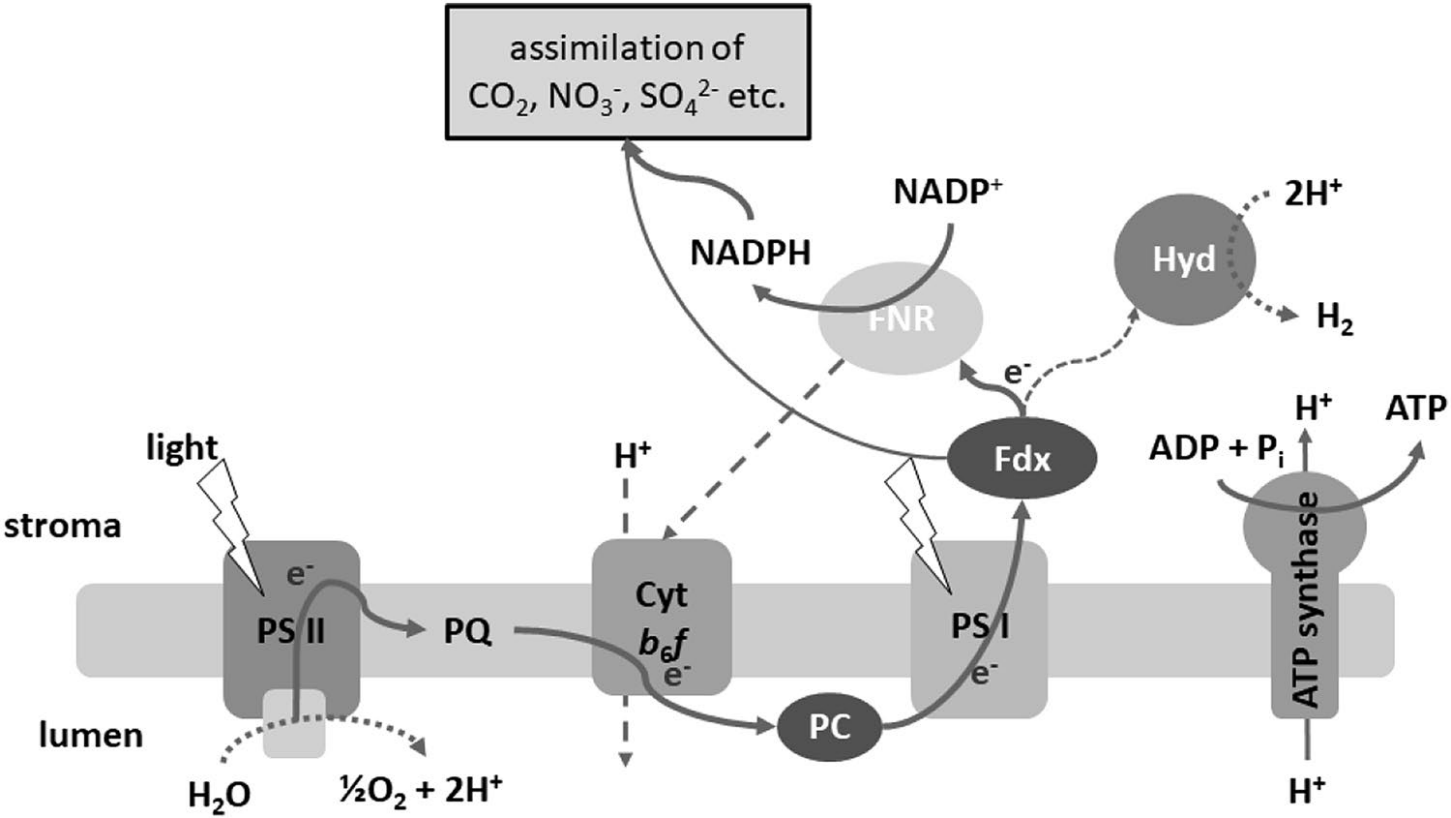
Schematic illustration of the photosynthetic electron transport in *C. reinhardtii*. Ferredoxin (primarily PetF) is the first stromal acceptor of photosynthetic electrons and delivers reductive energy to various metabolic pathways. Above all others to the FNR. *PS II* photosystem II, *PQ* plastoquinone, *Cyt b*_*6*_*f* cytochrome-b_6_f-complex, *PC* plastocyanin

Oxygenic photosynthesis has long been regarded as a blue-print for efficient light-driven synthetic processes. Its two photosystems are highly evolved molecular photovoltaic devices with excellent quantum yields. Photosystem I (PSI) achieves a low redox potential at its acceptor site, suitable for energy-intensive reductive syntheses, including the formation of molecular hydrogen [1]. In green algae like *Chlamydomonas reinhardtii*, natural photohydrogen production occurs due to the connection of the [FeFe]-hydrogenases HydA1 and HydA2 to the photosynthetic electron transport via the plant-type ferredoxin PetF [2, 3]. Ferredoxins are promising targets to manipulate the direction of electrons, as they are the first stromal mediators of the high-energy electrons derived from the absorption of light energy at PS1. However, due to the high affinity of the photosynthetic ferredoxin PetF for ferredoxin-NADP^+^ oxidoreductase (FNR), most of the electrons derived from the photosynthetic water splitting are used for the reduction of nicotinamide adenine dinucleotide phosphate (NADP^+^) and subsequently for CO_2_ fixation [4, 5] (Fig. 1). Therefore, utilizing photosynthetic organisms for the generation of biofuels such as H_2_ would require a rerouting of the photosynthetic electron flow.

Electron transfer interactions of natural [2Fe2S]-ferredoxins with their redox partners are very selective. Therefore, they systematically regulate electron flow, directing electrons to the necessary pathways under specified conditions [6–9]. Green microalgae like *Chlamydomonas reinhardtii* contain multiple plant-type [2Fe2S]-ferredoxin (Fdx) isoforms, all exhibiting the common sequence motif (Cx_4/5_Cx_2_Cx_n_), consisting of four [2Fe2S]-cluster-binding cysteine residues [7]. However, photosynthetic ferredoxin PetF, the most studied and abundant of them, acts as the main electron acceptor at PS1, donating reducing power to numerous redox partners like FNR, nitrite and sulfite reductase, pyruvate-ferredoxin-oxidoreductase (PFOR), as well as the two [FeFe]-hydrogenases (HydA1 and HydA2) [7, 10–13].

Efficiency of electron transfer between the FeS-clusters of redox partners is mainly based on three factors: differences in redox potential, distance of donor and acceptor FeS-cluster and protein–protein interactions [7]. For the latter, a first initial binding state, which consists of an ensemble of orientations, is supported by electrostatic interactions [14]. The final configuration is mostly supported by electrostatic as well as hydrophobic interactions [15].

As positions in the secondary ligand sphere of the FeS-cluster can contribute to both, determination of the redox potential [16] and complex formation assembly of the two interaction partners [8, 9, 17, 18], the redesign of already existing ferredoxins can be a difficult task. Furthermore, in a recent approach to manipulate the redox potential of PetF, we could reveal the complex nature of the protein framework, influencing the redox potential of the bound cofactor in multiple ways. The multitude of factors that can determine the influence of one particular position on the redox potential and intermolecular steric interactions makes the efficient design of PetF variants, suited to preferentially interact with the [FeFe]-hydrogenase, challenging [16].

Therefore, the identification of a natural ferredoxin capable of interacting with the hydrogenases, but not with FNR, might be a promising alternative to the redesign of PetF. Additionally, de novo designed [FeS]-proteins can serve as minimal models to reveal important structure function relationships of more complex natural proteins and to mimic their function [19–22]. Peptide maquettes and protein fragments can not only be used to understand their natural counterparts [23] but are as well a good starting point for the modular incorporation of different “redox modules” into artificial enzymes [24].

To screen for novel possibilities to direct photosynthetic electrons to specific enzymatic targets, we analyzed the interaction profiles of the two hydrogenases from *C. reinhardtii* with different plant-type ferredoxin isoforms, revealing remarkable differences in their affinity to the algal hydrogenases as well as to FNR. Furthermore, we constructed two minimal electron transfer peptides and tested their capability to direct electrons specifically towards the hydrogen producing enzymes. We identified the as yet uncharacterized ferredoxin Fdx7 as well as synthetic 4Fe4S cluster-binding peptide FbM1 to be a promising electron donor for HydA1, while interacting only poorly with FNR.

## Materials and methods

Unless stated otherwise, all chemicals and consumables were purchased from Sigma-Aldrich/Merck.

### Protein expression and purification

*Escherichia coli* strain BL21 (DE) *ΔiscR* [25], transformed with plasmids containing *E. coli* codon-optimized HYDA1 (NCBI accession number XP_001693376.1), HYDA2 (XP_001694503.1) sequences (devoid of the transit peptide encoding region) from *C. reinhardtii*, was used to recombinantly produce algal hydrogenase apo-enzymes (lacking the 2Fe_H_-subcluster of the active site, but containing the 4FeH-cluster). The expression and purification was conducted as described previously [26]. Cells were aerobically grown at 37 °C in lysogeny broth (LB, Sigma-Aldrich) medium supplemented with 0.1 M morpholinopropanesulfonic acid, 2 mM ammonium iron-citrate, and 5 g/L glucose until an OD_600_ of 0.35–0.5 was reached. They were transferred to an anoxic chamber (99% N2 1% H_2_ atmosphere, Coy), where 25 mM sodium fumarate was added. After 1 h and continuous stirring, 5 mM cysteine was added. The protein expression was initiated with 0.1 mM IPTG (Isopropyl β-d-1-thiogalactopyranoside). Cells were harvested by centrifugation after 16 h of incubation at 20 °C while stirring.

The *C. reinhardtii* sequences encoding ferredoxins CrPetF (XP_0016928 08.1), CrFdx2 (XP_001697912.1), CrFdx3 (XP_001691381.1), CrFdx8 (XP_001702123.2), and FNR (XP_001697352.1) were amplified from complementary DNA isolated out of total RNA from culture samples of *C. reinhardtii* strain CC-124. Genes of CrFdx7 (XP_001702098.1), CrFdx10 (XP_001703155.1), and CrFdx11 (XP_001695531.1) were optimized for *E. coli* codon-usage and purchased from Life Technologies GmbH (Darmstadt, Germany; www.thermofisher.com). In all cases, known or predicted sequences that may encode transit peptides were omitted. CrFdx sequences were cloned into vector pASK-IBA7, following a sequence encoding an N-terminal Strep-tagII and a factor Xa cleavage site, according to manufacturer’s recommendations (IBA Lifesciences, Göttingen, Germany; www.iba-lifesciences.com). Expression constructs for site-directed mutagenesis variants of CrFdx7 were generated following the procedure described in the Quik-Change-PCR manual from Agilent Technologies (Santa Clara, CA), using the corresponding 5′ overlapping mismatch primer pairs (Table S1). For the heterologous expression of FNR, the different Fdx isoforms and mutagenesis variants, electrocompetent *E. coli* BL21(DE3) *ΔiscR* cells were transformed using the respective expression construct. For ferredoxins, 4 L Vogel-Bonner (VB) medium was inoculated with overnight grown LB-preculture to an OD_550_ of 0.05 [27]; in case of FNR, LB medium was used instead of VB medium. Main cultures were grown at 37 °C in a shaking incubator (180 rpm) until an OD_550_ of 0.5 was reached. Gene expression was induced by adding anhydrotetracycline to a final concentration of 0.2 µg × mL^−1^, and expression cultures were kept for 16 h at 20 °C in a shaking incubator (180 rpm) until cell harvest by centrifugation (20 min, 9000*g*, and in 4 °C).

Cell pellets were resuspended 0.1 M Tris–HCl (pH 8) (in case of the hydrogenases, 2 mM sodium dithionite (NaDT) was added to all buffers and all work was performed under anaerobic conditions). Cell disruption was carried out by ultrasonication, and the resulting cell lysate was centrifuged at 165,000*g* for 1 h at 4 °C. The supernatant was filtered using sterile syringe filters (0.2 mm pore size; SARSTEDT, Newton, NC). The recombinant proteins were purified via affinity chromatography using StrepTag Superflow high-capacity gravity flow columns (IBA Lifesciences), according to the manufacturer's recommendations, and concentrated using Amicon Ultracel filters (Merck Millipore, Burlington, MA) with a 10 kDa cutoff in case of ferredoxins and 30 kDa cutoff in case of FNR and hydrogenases. For the hydrogenases, protein concentration was determined by Bradford assay [28]. For the ferredoxins, protein concentration was determined via UV–Vis spectroscopy (BioPhotometer D30 from Eppendorf, Hamburg, Germany www. eppendorf.com) at 420 nm applying the Beer-Lambert Law and using a molar extinction coefficient of 9.7 mM^−1^ × cm^−1^ [29]. The FNR concentration was determined according to the specific absorption maximum of its cofactor flavin adenine dinucleotide (FAD), with a molar extinction coefficient of 9.4 mM × cm^−1^ at 457 nm [30]. Until further use, all proteins were stored at − 80 °C in 0.1 M Tris–HCl (pH 8).

### In vitro maturation of [FeFe] hydrogenases

Hydrogenases with fully assembled active sites were generated using the previously described in vitro maturation [31]. Apo-proteins (lacking the 2Fe_H_ moiety after recombinant expression) were incubated on ice for 1 h with a tenfold molar excess of 2Fe_H_ cofactor mimic [2Fe_2_ [µ-(SCH 2)_2_NH](CN)_2_(CO)_4_]^2−^ [32] in 0.1 M potassium-phosphate buffer (K_2_HPO_4_/KH_2_PO_4_ pH 6.8, KPI). Resulting holo-proteins were purified from excess 2Fe_H_ by size exclusion chromatography (NAP 5 column, GE healthcare).

### FeS-cluster reconstitution of peptides

The peptides used in this work were synthetized by Thermo Scientific Custom Peptide Synthesis Service (www.thermofisher.com). Peptides were solved in 50 mM HEPES (4-(2-hydroxyethyl)-1-piperazineethanesulfonic acid) buffer pH 8 to a concentration of 500 µM in a total volume of 1 mL under anaerobic conditions. F_B_M-1 samples were incubated with 2 mM DTT (dithiothreitol) for 1 h on ice. A fourfold molar excess of NaS_2_ and FeCl_3_ was added to the samples in case of F_B_M-1 and a twofold molar excess of NaS_2_ and FeCl_3_ was added to PM-1 samples. After 30 min of incubation at room temperature, all peptide samples were purified by size exclusion chromatography using Sephadex G-10 column material (GE healthcare). Subsequently, the peptide samples were concentrated using Amicon Ultracel filters with a 3 kDa cutoff and were stored in 50 mM HEPES pH 8 at − 80 °C until further use.

### Fdx:Hyd interaction: in vitro hydrogenase activity assay

To determine the H_2_ production activity with ferredoxins and FeS-cluster-binding peptides as an electron mediator, 80 ng holo-hydrogenase was incubated for 30 min at 37 °C in sealed 3 mL headspace vials (Suba) containing a reaction mix of 300 µL 0.1 M KPI pH 6.8 supplemented with 10 mM NaDT as sacrificial electron donor and 50 µM ferredoxin as electron mediator. Prior to incubation, the headspace of the vessels was degassed with 100% argon. After incubation, a 400 µL sample of the headspace was analyzed via gas chromatography (GC-2010, Shimadzu). Hydrogenase activity resulting from reduction by NaDT alone was deducted from results by subtracting activity values gained from samples containing no electron mediator from the results obtained with ferredoxin and [FeS] cluster-binding peptides. All measurements were carried out using ferredoxin/[FeS] peptide samples derived from two independent protein preparations.

### Fd:FNR interaction: in vitro cytochrome c reduction assay

The ability to reduce certain ferredoxins by FNR was examined by the cytochrome c reduction test [33]. In this assay, FNR is reduced by NAPDH and therefore reduces ferredoxin, which finally reduces cytochrome c. The reduction of cytochrome c can be measured by an increase of the absorption at 550 nm. Samples contained 100 µM NADPH, 2 U × mL^−1^ glucose-6-phosphate dehydrogenase (G-6-P-DH), 5 mM glucose-6-phosphate (G-6-P), 40 nM FNR, 5 µM ferredoxin, and 100 µM cytochrome c from bovine heart in a total volume of 300 µL 50 mM Tris–HCl (100 mM NaCl, pH 7.5). The reaction was started by addition of ferredoxin, and the absorption shift at 550 nm was followed spectrophotometrically for 160 s. The reaction velocity was calculated by linear regression of the first 30 s of reaction for PetF, Fdx2, Fdx10, and Fdx11. In case of Fdx3, Fdx7, and Fdx8, the complete timescale of 160 s was taken in account. All measurements were carried out using ferredoxin/[FeS] peptide samples derived from two independent protein preparations.

### Light-driven hydrogen production and competition assay

To determine the light-driven H_2_ production, 50 nM HydA1 was combined with 10 µM ferredoxin. The total volume of 200 µL contained 40 mM EDTA as a sacrificial electron donor and 200 µM proflavine (acridine-3,6-diamine) as a photosensitizer in 100 mM potassium phosphate pH 6.8, supplemented with 0.1 mM sodium dithionite and 3 mM NaNO_3_. To determine the H_2_ production efficiency of HYDA1 under competitive conditions, 50 nM FNR and 2 mM NADP^+^ were added. For stabilizing the level of NADP^+^ and thus the competitive efficiency of the FNR during H_2_ production, 0.1 U of nitrate reductase (NAR) from *Aspergillus niger* was further included. All samples were prepared under anoxic conditions in 2 mL tubes and sealed anaerobically). After degassing the headspace of the vessels with 100% argon, the samples were light-exposed (1500 µmol photons m^−2^ × s^−1^) under constant shaking at 37 °C. H_2_ production was determined after 30 min by analyzing 400 µL of the headspace via gas chromatography (GC-2010, Shimadzu). Hydrogenase activity resulting from reduction by NaDT alone was deducted from results by subtracting activity values gained from samples containing no electron mediator from the results obtained with ferredoxin and [FeS] cluster-binding peptides. All measurements were carried out using ferredoxin/[FeS] peptide samples derived from two independent protein preparations.

## Results and discussion

Twelve Fdx paralogs have been identified in the *C. reinhardtii* genome [7]. While, for lately discovered ferredoxin isoforms CrFdx7–12, no physiological function could be assigned yet, several physiological functions were presumed for CrFdx2–6 based on studies using transcript data, yeast two-hybrid screens, in vitro activity assays and affinity pulldown assays [6, 34–39]. Some of the until now uncharacterized ferredoxin isoforms (Fdx 7–9) have been predicted to be localized in the chloroplast [7]. This prompted us to test the capability of sodium dithionite reduced ferredoxins (Fdx 7, 8, 10 and 11) to serve as electron donors for the *Chlamydomonas* hydrogenases HydA1 and HydA2. Since former yeast-2-hybrid studies [6], suggested a different interaction pattern of HydA1 and HydA2 with Fdx3, respectively, we also sought to test Fdx3 as electron source. Fdx2 and PetF, well characterized for both hydrogenases, served as control [40, 41].

**Fig. 2:**
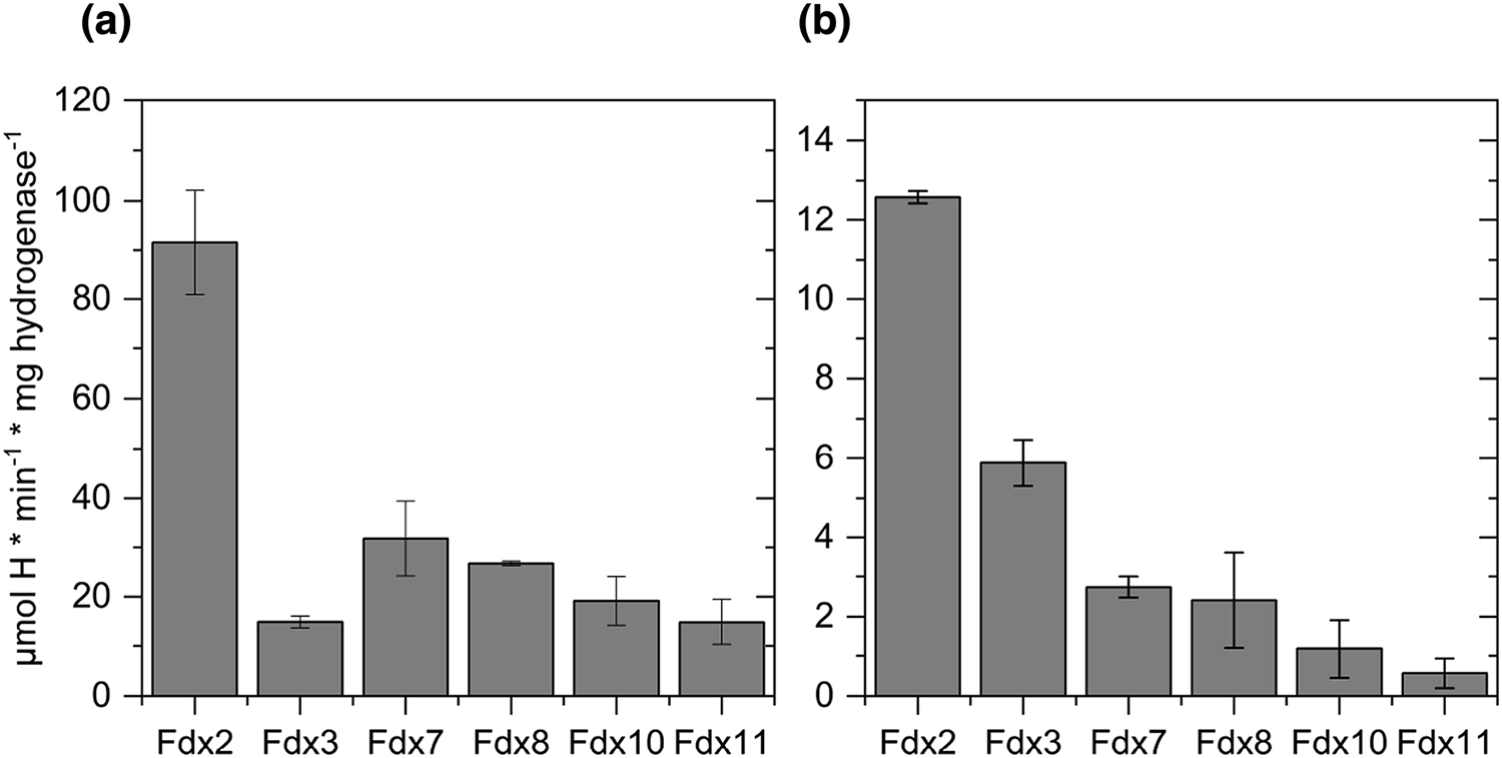
Ferredoxin-dependent hydrogenase activities of **a** HydA1 and **b** HydA2. H_2_ production rates of purified algal HydA1 and HydA2 with algal [2Fe-2S]-ferredoxins (50 μM, reduced with 10 mM sodium dithionite). The averages of two biological replicates are shown; *error bars* indicate the standard deviation

To ensure the consistency of data derived from in vitro H2 production assays, HydA1 and HydA2 were tested for their methyl viologen- and PetF-dependent hydrogen evolution activities (Fig. S1). These results were in agreement with previously published data [13, 41]. All of the ferredoxins tested here were able to reduce HydA1 for hydrogen evolution. With 225 ± 18 µmol H2 × min^−1^ × mg^−1^, the highest hydrogen production was achieved using PetF as an electron donor for HydA1 (Fig. S1), while the use of Fdx2 in this assay yielded about 40% of the hydrogen production obtained with the use of PetF (Fig. 2a). With 14% of PetF-dependent hydrogen production, Fdx7 showed the highest rates among the remaining Fdx isoforms. While Fdx8-dependent hydrogen production was in a similar range as values measured with Fdx7, Fdx10, and Fdx11 were only half as efficient in reducing HydA1 as Fdx7. The lowest hydrogen production rate was observed with Fdx3.

While hydrogen production activities of HydA2 were generally lower than those of HydA1, PetF, and Fdx2 were the most efficient electron donors to HydA2 as well, hydrogen production rates achieved with PetF were at 63,9 ± 7.7 µmol H2 × min^−1^ × mg^−1^ (Fig. S1) and 12.6 ± 0.2 µmol H2 × min^−1^ × mg^−1^ for Fdx2 (Fig. 2b). Fdx3 was the third most efficient electron donor with 10% of the hydrogen production measured with PetF. These results are similar to the findings of former studies, assigning a moderate interaction for HydA2 and Fdx3 in their yeast-2-hybrid study [6]. It is interesting to note that the midpoint potential of Fdx3 and Fdx7 was recently determined to be in the range of Fdx2 in case of Fdx7 (− 321 mV at pH 7.5) and even more positive in case of Fdx3 (− 250 mV). Physiological studies suggested that so-called root-type ferredoxin CrFdx2 is involved in reactions with a comparatively more positive midpoint potential, e.g., in nitrite reduction [42]. On the other hand, H_2_ production, with the H^+^/H_2_ half-cell couple having a standard biochemical midpoint potential of − 410 mV, is expected to require electron donors with much more negative midpoint potentials. Besides midpoint redox potentials, electrostatic interactions between the surfaces of the redox partners are crucial for the interaction between ferredoxins and hydrogenases [3, 7, 18]. The relatively low H_2_ production activities using Fdx7 as electron donor for HydA1 show that even though its midpoint potential is not suitable to efficiently drive the reaction, Fdx7 is able to form a functional complex with HydA1, allowing electron transfer and thus hydrogen production.

However, reduction potentials are sensitive to concentrations of reactants and products according to the Nernst equation, and therefore a high excess of reduced sodium dithionite in our in vitro assays might only be suitable to identify proteins that interact with the hydrogenases, rather than being suitable electron donors under in vivo conditions. On the other hand, single exchanges lowering the redox potential of Fdx2 by site-directed mutagenesis significantly increased in vitro H_2_ production and lowered NADPH photoproduction [40].

This study, together with our recent manipulation of the redox potential of PetF, shows that the midpoint potential of ferredoxins can be tuned by single-point mutations [16]. As the manipulation of protein–protein interactions, especially with respect to the influence of one single exchange to the variety of redox partners of PetF, can be a more complicated task, the identification of a natural ferredoxin with a suitable binding affinity to redox enzymes of interest is a good starting point for the rational design of electron donors, tailored to specifically deliver reducing power to, for example, the hydrogenases instead of FNR.

**Fig. 3:**
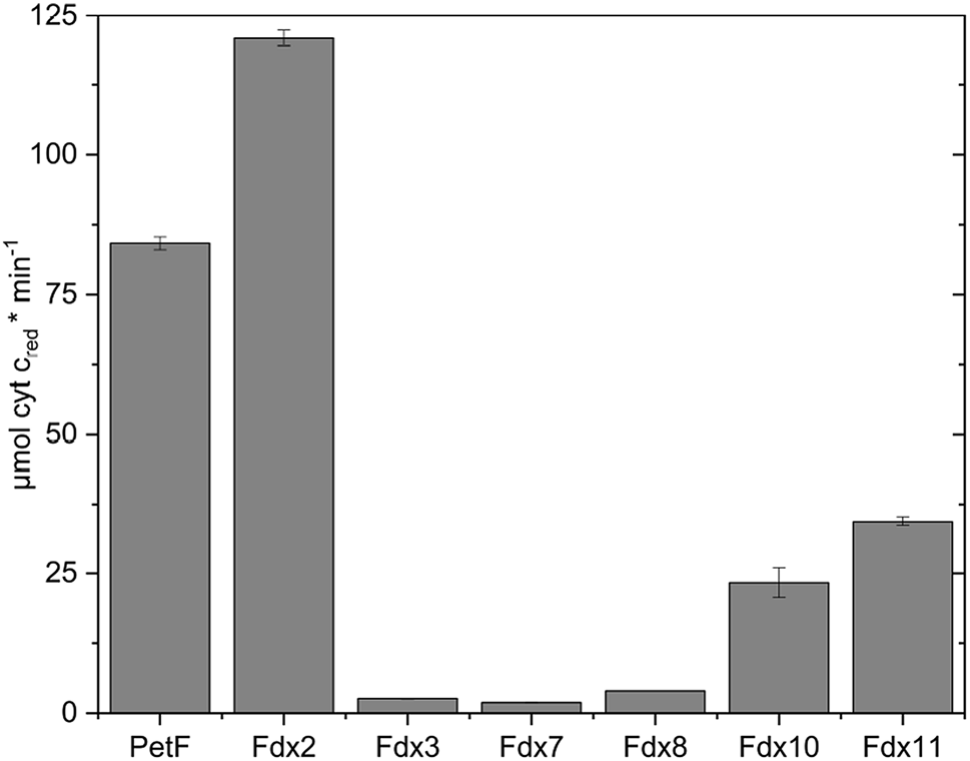
Electron transfer between FNR and Fdx indirectly measured by NADPH-dependent cytochrome c reduction. 40 nM FNR, 5 µM ferredoxins (from *C. reinhardtii*), and 100 µM cytochrome c were incubated in the presence of 100 µM NADPH. Cyt c reduction was measured photometrically at 550 nm. Error bars depict the mean ± standard deviation for 3–5 measurements from two biological replicates

Prior studies suggested that while PetF and Fdx2 were able to interact with FNR, Fdx3 was not able to drive NADPH production [6, 40]. Identifying Fdx7 and Fdx8 as potential electron donors for HydA1, we sought to investigate the interaction pattern of the ferredoxin isoforms with FNR alone. With PetF as electron mediator between FNR and cyt c, the initial reaction speed was measured at 84.2 ± 1.2 µM cyt c × min^−1^ (Fig. 3). The highest reaction speed was observed with Fdx2 at 121.1 ± 1.4 µM cyt c × min^−1^. This is in line with former observations, comparing the kinetics of the electron transfer of FNR with PetF and Fdx2, respectively [42].

Only very low electron transfer rates where measured for Fdx3, Fdx7, and Fdx8. It was already shown that Fdx3 did not drive NADPH photoproduction at detectable rates [6]. Fdx10 and Fdx11 both showed higher cyt c reduction rates with 23.3 ± 2.7 µM cyt c × min^−1^ and 34.4 ± 0.8 µM cyt c × min^−1^, respectively.

Comparing the results of our in vitro studies, ferredoxins 7 and 8 were both able to transfer electrons to HydA1, while their interaction with FNR seemed to be impaired. Given the competition for electrons between FNR and HydA1 during H_2_ production, we aimed to obtain informations about the performance of the newly identified ferredoxins in the presence of both enzymes in one assay, mimicking the competition of HydA1 and FNR for reduced ferredoxin as it appears in the chloroplast, e.g., during algal H_2_ photoproduction [43].

**Fig. 4:**
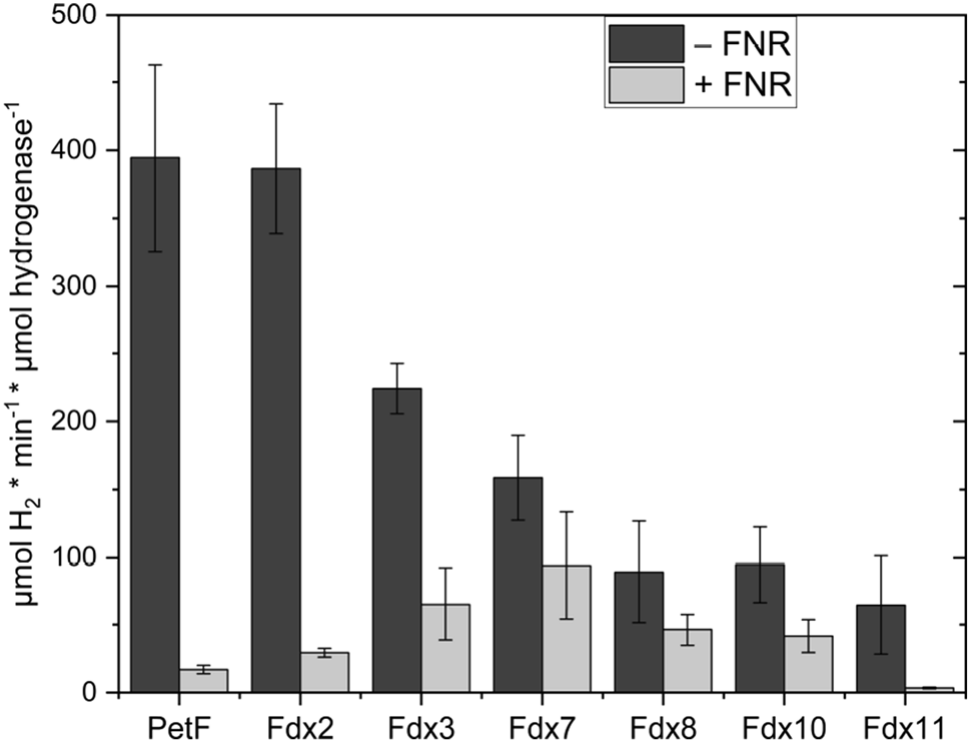
Rates of light-dependent H_2_ production were determined for HydA1 with selected Fdx isoforms in the absence (*dark gray*) and in the presence (*light gray*) of FNR. *Error bars* depict the mean ± standard deviation for 2–4 measurements from two biological replicates

After individual assessment of the hydrogenase and FNR interactions of different ferredoxin isoforms, in vitro H_2_ photoproduction by HydA1 was measured in competition with FNR from *C. reinhardtii*. In this assay, EDTA was used as sacrificial electron donor and proflavine as photosensitizer [8]. The proflavine reduced Fdx is either oxidized by HydA1, resulting H_2_ evolution or by FNR, resulting in NADPH production. Hydrogen production was measured in samples with and without addition of FNR to compare the impact on hydrogen production by the presence of FNR. In presence of FNR, H_2_ photoproduction is reduced to 3–6% when PetF or Fdx2 are used as electron mediators (Fig. 4). In case of Fdx8 and Fdx10, hydrogenase activity in the presence of FNR was reduced to 52% and 43%, while the H_2_ evolution rates under non competitive conditions reached only about 20% of the activity obtained with PetF as electron mediator. The use of Fdx3 and Fdx7 in the absence of FNR resulted in about 50% H_2_ photoproduction compared to PetF and Fdx2. In case of Fdx3, the presence of FNR reduced the hydrogen production activity to 29%. Fdx7 samples showed the highest H_2_ production in presence of FNR as competitor for HydA1 by remaining 59% of activity compared to activities reached under noncompetitive conditions.

The results of this direct competition assay confirmed the promising results of Fdx7 with FNR and HydA1 alone. Identifying Fdx7 as a suitable target for rational protein design, we tried to increase the effect of the favourable Fdx:Hyd complex formation by introducing four single exchanges in the expected area of the binding interface. Mutation sites were chosen based on earlier studies that identified certain amino acid residues of PetF to be important for the interactions with hydrogenase and FNR or the manipulation of the midpoint potential, respectively. In PetF, D56, E122, and F93 were proposed to be essential for the final Fdx:Hyd complex orientation, while F93 and Y126 were suggested to be essential for the lower midpoint potential [3, 7, 11]. Our recent study identified serine 43 as another midpoint determining factor. However, neither the introduction of functional groups which were expected to lower the midpoint potential of Fdx7 based on the results of PetF (Fdx7 variants S38E, A72F, and W106Y), nor the introduction of negative net charge to further stabilize the Fdx7:Hyd complex formation (I102E) led to significant changes during H_2_ production (Fig. S2), cyt c reduction (Fig. S3) or in the FNR-hydrogenase competition assay (Fig. S4).

These results show that manipulating the catalytic performance by adjusting the electron transfer process is complicated, and though Fdx7 still seems an interesting target for further studies to drive the electron flow to the hydrogenases, sequence information from the well-studied PetF cannot be easily transferred to other Fdx isoforms. One of the reasons for this, apart from the relatively low-sequence identity of Fdx7 and PetF (24%), might be that the manipulation of a single feature of the about 100 aa covering peptide sequence is rather complicated: the binding interface of plant-type ferredoxins with their interaction partners is in direct vicinity to the partly surface exposed FeS-cluster [44]. Therefore, exchanging a position close to the FeS-cluster could affect protein folding, midpoint potential, and binding affinity simultaneously. To further simplify the protein surrounding the FeS-cluster as an electron donor for HydA1, and therefore make it a target to be easier manipulated, the utilization of short FeS containing peptides seems to be promising. So-called peptide maquettes have been shown to achieve redox functions and provide electrons to, for example, heme containing metallo-enzymes [45]. We therefore chose 16 aa peptide maquette F_B_M1 known to bind a redox active 4Fe4S cluster [19] to test its capability to (1) deliver electrons to HydA1 or (2) to receive electrons from FNR and reduce cyt c as a first assessment of its interaction with FNR. Additionally, we designed PM-1, a 22 aa peptide maquette based on the *Chlamydomonas* PetF sequence (Table S2). UV–Vis spectroscopy of reconstituted peptides indicated the successful reconstitution of FeS-clusters (Fig. S5).

**Fig. 5:**
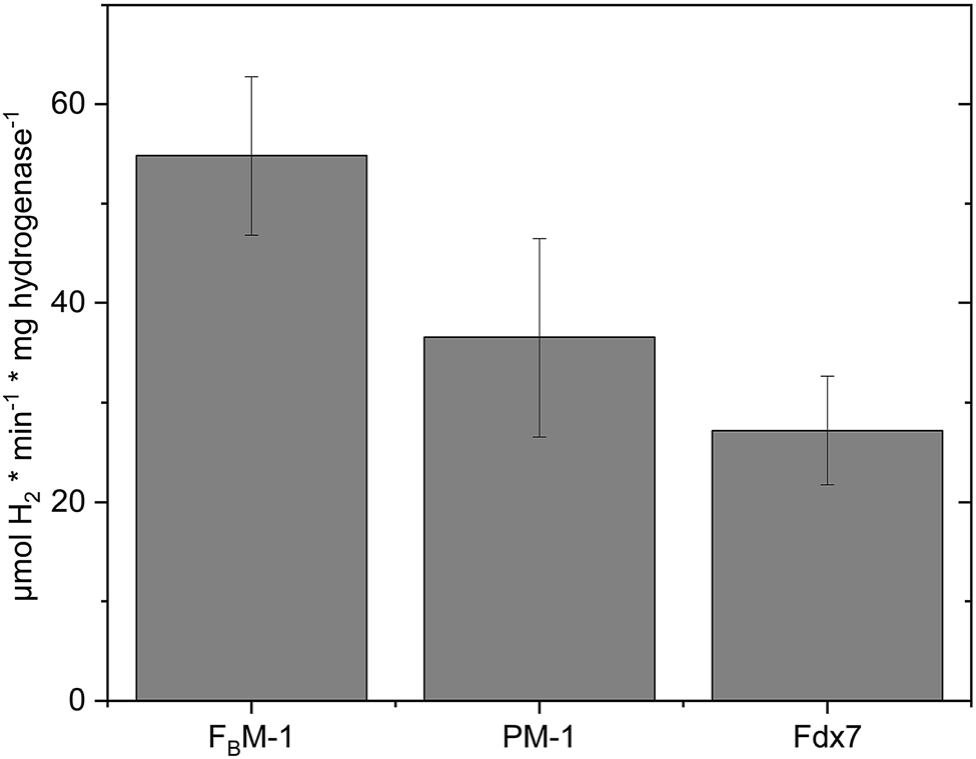
H_2_ production rates of purified algal HydA1 with different electron mediators (50 μM, reduced with 10 mM sodium dithionite). The averages of two biological replicates are shown; *error bars* indicate the standard deviation

In vitro H_2_-production assays showed the ability of reconstituted peptides to reduce HydA1 for H_2_ evolution, yielding specific activities even higher than those with Fdx7 (54.8 ± 9 µmol H_2_ × min^−1^ × mg^−1^ for F_B_M-1, 36.5 ± 10 µmol H_2_ × min^−1^ × mg^−1^ for PM-1, Fig. 5).

**Fig. 6:**
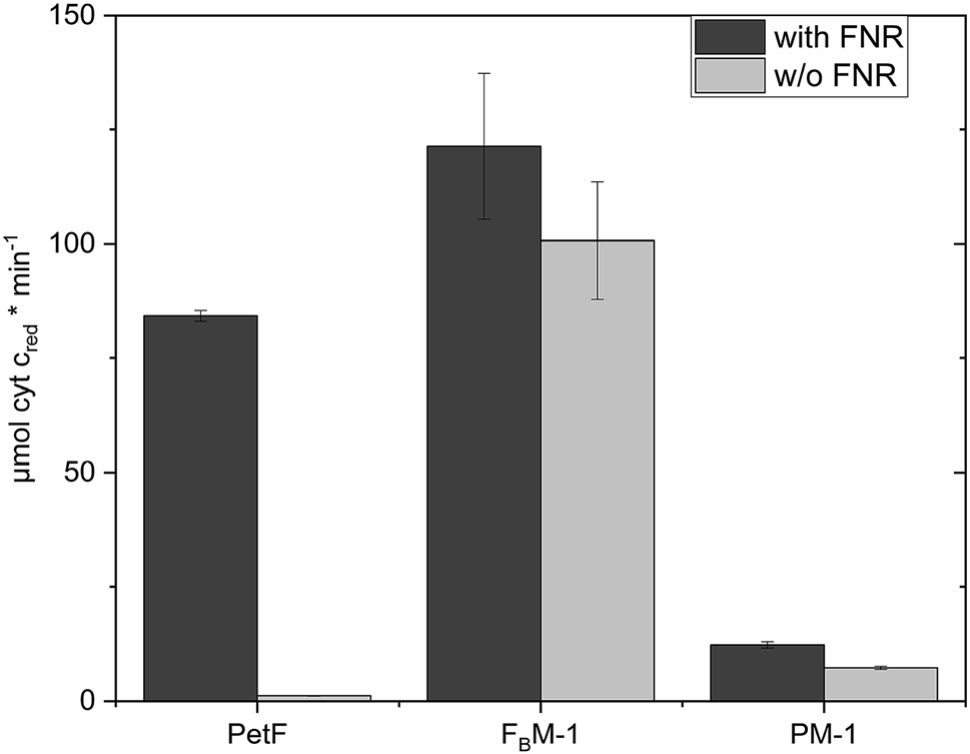
Electron transfer between FNR and Fdx indirectly measured by NADPH-dependent cytochrome c reduction. 40 nM FNR, 5 µM electron mediator, and 100 µM cytochrome c were incubated in the presence of 100 µM NADPH. Cyt c reduction was measured photometrically at 550 nm. Results from samples with addition of FNR (*dark gray*) and without addition of FNR (*light gray*). *Error bars* depict the mean ± standard deviation for 2–3 measurements two biological replicates

The interaction of the reconstituted peptides F_B_M-1 and PM-1 with FNR was investigated by the cyt c reduction assay, as well. The conversion rates assessed with PM-1 as electron mediator were 12.3 ± 0.7 µmol cyt c × min^−1^ and 121.3 ± 15.9 µmol cyt c × min^−1^ for F_B_M-1 (Fig. 6). The reaction processes differed from those of the ferredoxin samples by showing very high cyt c reduction activity in the very first seconds of reaction (Fig. S6). For this reason, additional control reactions without FNR were introduced. While only minor cyt c reduction rates of 1.2 ± 0.1 µmol cyt c × min^−1^ were measured in non FNR containing PetF samples, cyt c reduction rates in absence of FNR were reduced by 40% in PM-1 samples and only 17% in F_B_M-1 samples. This observation led to the assumption that the reconstituted peptides can be reduced by NADPH.

Concludingly, we have identified electron donors, which prefer the interaction with HydA1 over FNR (Fdx7, Fdx8), and have created minimal electron transfer units, which might be reduced by NADPH alone and are able to deliver electrons to HydA1. Other studies have found that FeS-cluster containing peptides are redox active [19, 46–50] and can reduce natural proteins [51]. Here, we could show that specifically designed peptides, reconstituted with FeS-clusters, can even replace natural ferredoxin as electron shuttles in more sophisticated in vitro experiments. Future studies may focus on the further manipulation of the midpoint potential of Fdx7 to further increase the H_2_ production rates of HydA1 with Fdx7, either aim to integrate peptide maquettes or Fdx variants into algal cells to redirect electron flow towards HydA1 for in vivo H_2_ production.

## Supplementary Information

Below is the link to the electronic supplementary material.

Supplementary file1 (PDF 390 KB)
