# Supplementary material for: Changing the tracks: screening for electron transfer proteins to support hydrogen production
Source: J Biol Inorg Chem. 2022 Aug 29;27(7):631–40. doi: 10.1007/s00775-022-01956-1 (PMC9569306; doi:10.1007/s00775-022-01956-1)
Supplement: Supplementary file 1 — Supplementary file1 (PDF 390 KB) [file 775_2022_1956_MOESM1_ESM.pdf]

Electronic Supplementary Information

Changing the tracks: screening for electron transfer proteins to support hydrogen production

Alexander Günzel[1], Vera Engelbrecht[1], Thomas Happe[1]

✉ Thomas Happe
thomas.happe@rub.de

[1] Faculty of Biology and Biotechnology, Photobiotechnology, Ruhr-University Bochum, Universitätsstraße 150, 44801 Bochum, Germany

Table S1 QuikChange primers used for site-directed mutagenesis of CrFdx7. Mismatch positions are presented in bold.

Primer name	Nucleotide sequence
Fdx7_S38E_fw	GATTTCATGGATGTTGCA**GAG**CGTTGTAAAG
Fdx7_S38E_rv	GAATATCTGCTTTACAACG**CTC**TGCAACATCC
Fdx7_A72F_fw	TGCAGAAAGCGGTGAA**TTC**CGTGAAGCAG
Fdx7_A72F_rv	TTACCTGCTTCACG**GAA**TTCACCGCTTTCTGC
Fdx7_I102E_fw	GTTGGTATGATGGCA**GAG**GATCAGGTTTGG
Fdx7_I102E_rv	CCAAACCTGATC**CTC**TGCCATCATACCAAC
Fdx7_W106Y_fw	GGCAATTGATCAGGTTT**AC**GGTCAAGATGG
Fdx7_W106Y_rv	GGTATCCCAACCATCTTGACC**GT**AAACC

Table S2 Amino acid sequences and molecular weight of oligopeptides used for preparation of [FeS] cluster containing peptides.

Peptide name	Amino acid sequence	Molecular weight g / mol
F_BM-1	YDTCIGCTQCKPECPW	1847.12
PM-1	PYSCRAGACSSCAGSGGALTCV	2021.31

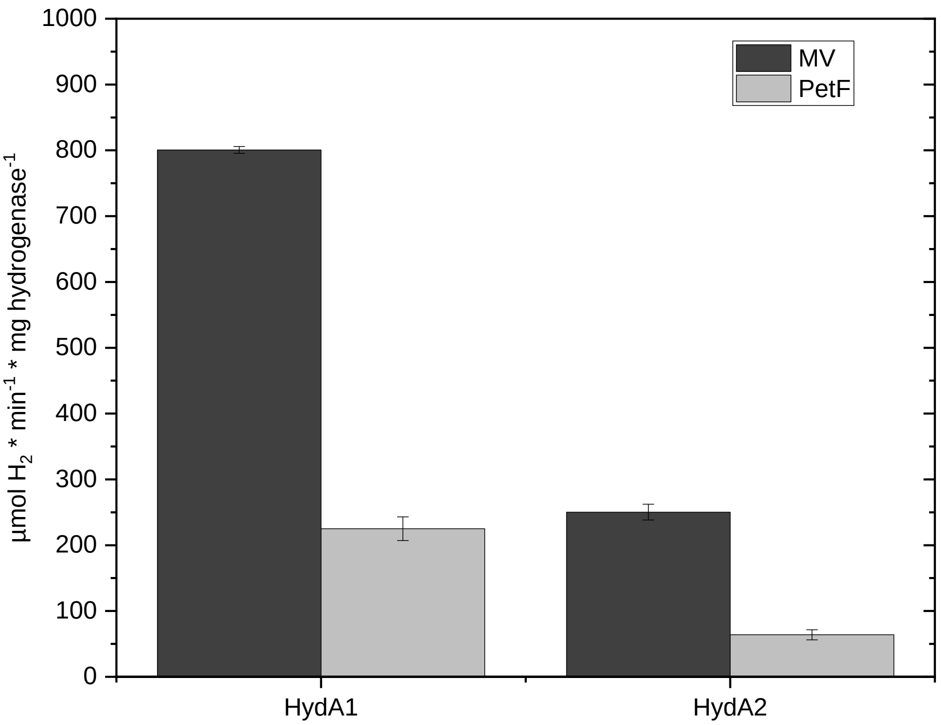

Fig. S1 Methylviologen- and PetF- dependent hydrogenase activities. H_2 production rates of purified algal HydA1 and HydA2 methylviologen (10 mM reduced with 100 mM sodiumdithionite) shown in *dark grey* and with [2Fe-2S]-ferredoxin PetF (50 µM, reduced with 10 mM sodium dithionite) shown in *light grey*. The averages of two technical replicates are shown, error bars indicate the standard deviation

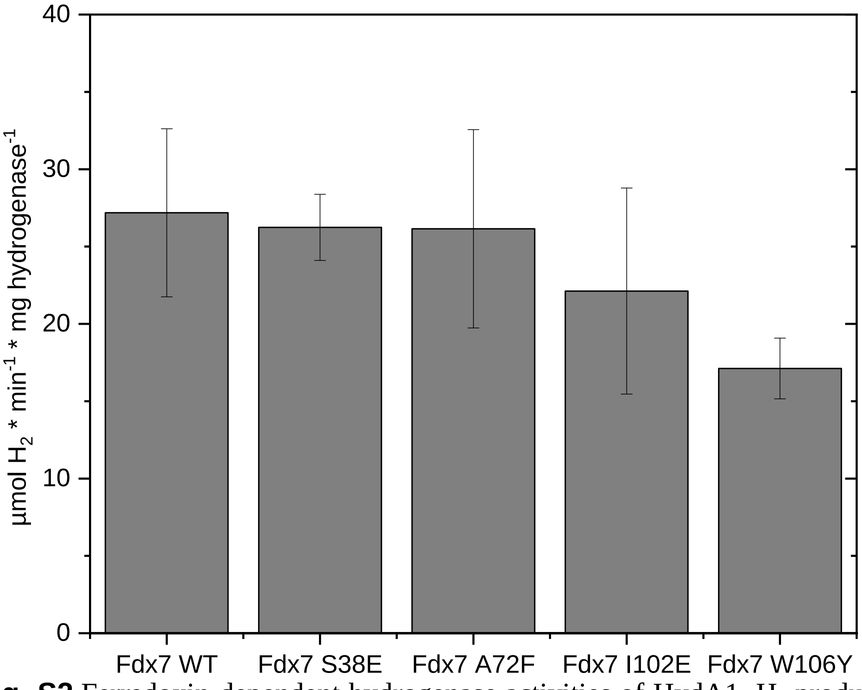

Fig. S2 Ferredoxin-dependent hydrogenase activities of HydA1. H_2 production rates of purified algal HydA1 with algal [2Fe-2S]-ferredoxins (50 µM, reduced with 10 mM sodium dithionite). The averages of two biological replicates are shown, error bars indicate the standard deviation

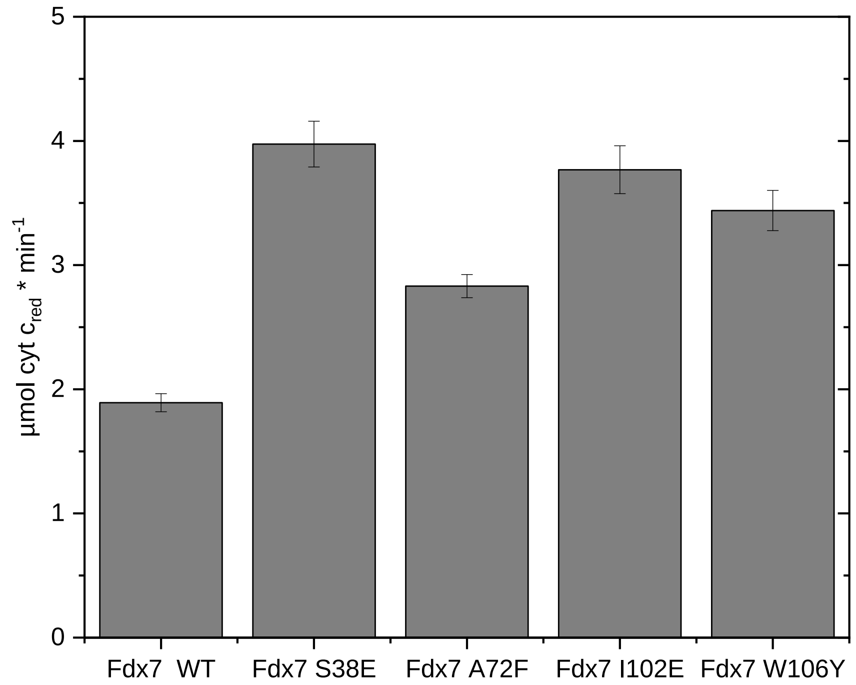

Fig. S3 Fdx:FNR interaction: Electron transfer between FNR and Fdx indirectly measured by NADPH-dependent cytochrome c reduction. 40 nM FNR, 5 µM ferredoxin (both from *C. reinhardtii*) and 100 µM cytochrome c were incubated in the presence of 100 µM NADPH. Cyt c reduction was measured photometrically at 550 nm. Error bars depict the mean ± standard deviation for measurements from two biological replicates.

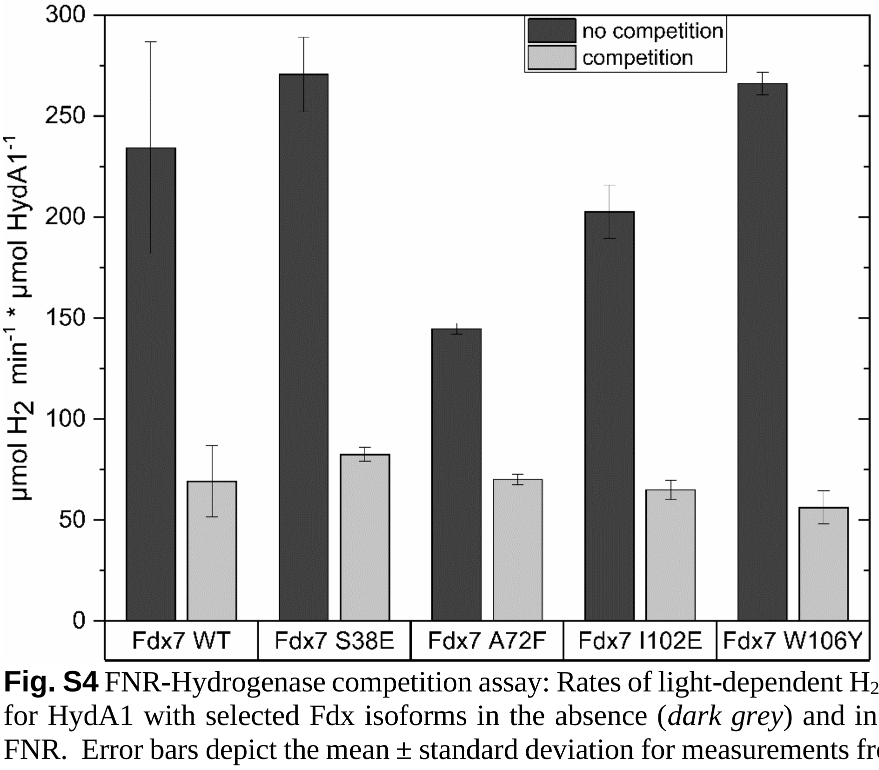

Fig. S4 FNR-Hydrogenase competition assay: Rates of light-dependent H_2 production were determined for HydA1 with selected Fdx isoforms in the absence (*dark grey*) and in the presence (*light grey*) of FNR. Error bars depict the mean ± standard deviation for measurements from two biological replicates.

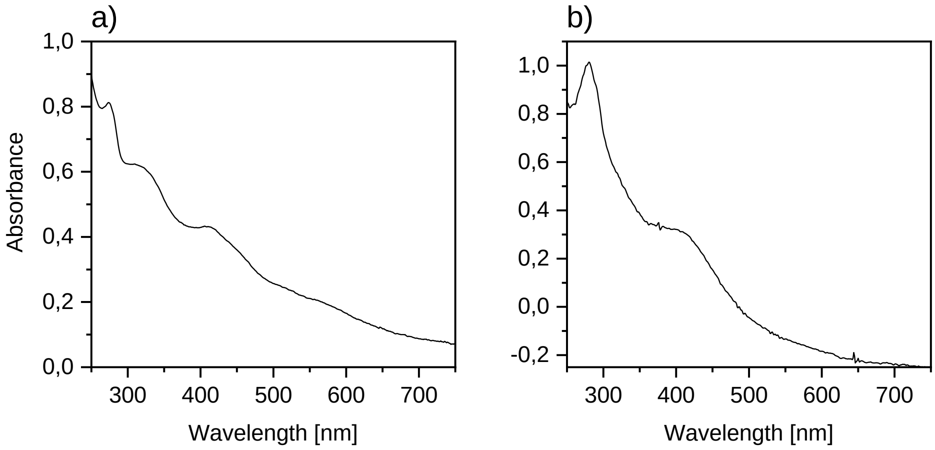

Fig. S5 UV-vis spectra of reconstituted peptides. **a)** PM-1 **b)** F_BM-1.

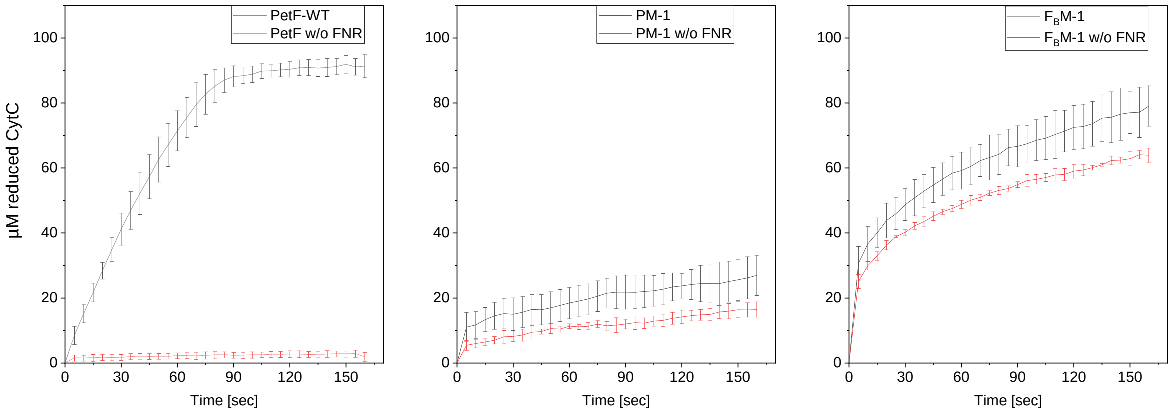

Fig. S6 Peptide:FNR interaction: Time resolved display of results presented in Fig. 6. Electron transfer between FNR and Fdx indirectly measured by NADPH-dependent cytochrome c reduction. 40 nM FNR, 5 µM electron mediator and 100 µM cytochrome c were incubated in the presence of 100 µM NADPH. Cyt c reduction was measured photometrically at 550 nm. Error bars depict the mean ± standard deviation for 2-3 measurements from 2 independent preparations.

Table S3 Values of the hydrogen production measurements presented in this work.

electron mediator	1st measurement	2nd measurement	3rd measurement	4th measurement	mean value	standard deviation (+/-)
**Figure 2a – Fdx-dependent HydA1 activity [µmol H_2 *min^{-1} *mg^{-1}]**						
Fdx2	80.92	102.07			91.49	10.58
Fdx3	13.82	16.17			14.99	1.17
Fdx7	39.42	24.14			31.78	7.64
Fdx8	27.27	26.4			26.84	0.44
Fdx10	24.03	14.31			19.17	4.86
Fdx11	19.52	10.31			14.92	4.6

**Figure 2b – Fdx-dependent HydA2 activity [µmol H_2 *min^{-1} *mg^{-1}]**						
Fdx2	12.75	12.44			12.59	0.15
Fdx3	6.48	5.31			5.89	0.58
Fdx7	2.48	3			2.74	0.26
Fdx8	1.19	3.63			2.41	1.22
Fdx10	0.45	1.91			1.18	0.73
Fdx11	0.93	0.2			0.57	0.37
**Figure 4 – competition assay HydA1 w/o FNR [µmol H_2 *min^{-1} *µmol^{-1}]**						
PetF	392.42	507.12	349.92	328.53	394.5	68.97
Fdx2	454.08	404.9	328.66	357.99	386.41	47.6
Fdx3	252.12	229.3	206.38	208.81	224.15	18.44
Fdx7	208.65	144.95	156.81	124.3	158.68	31.11
Fdx8	84.25	150.94	71.77	49.78	89.19	37.73
Fdx10	130.02	109.75	83.46	55.72	94.74	27.93
Fdx11	58.4	118.53	67.42	15.39	64.93	36.66
**Figure 4 – competition assay HydA1 with FNR [µmol H_2 *min^{-1} *µmol^{-1}]**						
PetF	17.3	18.83	11.78	19.89	16.95	3.12
Fdx2	26.93	25.21	32.72	32.34	29.3	3.29
Fdx3	92.06	89.03	53.84	27.11	65.51	26.78
Fdx7	120.01	123.99	37.57		93.86	39.83
Fdx8	47.47	60.18	31.7		46.45	11.65
Fdx10	45.53	53.93	25.21		41.56	12.06
Fdx11	2.9	4.03			3.46	0.57
**Figure 5 – Fdx-dependent HydA1 activity [µmol H_2 *min^{-1} *mg^{-1}]**						
F_BM-1	62.8	46.84			54.82	7,98
PM-1	26.55	46.49			36.52	9.97
Fdx7	32.61	21.75			27.18	5.43
**Figure S1 – HydA1 and HydA2 activity [µmol H_2 *min^{-1} *mg^{-1}]**						
**HydA1**						
MV	805.72	795.46			800.59	5.13
PetF	207.07	243.03			225.05	17.98
**HydA2**						
MV	262.33	238.21			25.27	12.06

PetF	56.29	71.6		63.95	7.65
**Figure S2 – Fdx-dependent HydA1 activity [µmol H_2 *min^{-1} *mg^{-1}]**					
Fdx7 WT	32.61	21.75		27.18	5.43
Fdx7 S38E	24.1	28.38		26.24	2.14
Fdx7 A92F	32.56	19.73		26.15	6.41
Fdx7 I102E	15.46	28.78		22.12	6.66
Fdx7 W106Y	19.08	15.16		17.12	1.96
**Figure S4 – competition assay HydA1 w/o FNR [µmol H_2 *min^{-1} *µmol^{-1}]**					
Fdx7 WT	181.8	286.57		234.19	52.39
Fdx7 S38E	289.10	252.07		270.58	18.52
Fdx7 A92F	141.91	147.4		144.62	2.74
Fdx7 I102E	189.41	215.82		202.62	13.21
Fdx7 W106Y	260.41	271.67		266.04	5.63
**Figure S4 – competition assay HydA1 with FNR [µmol H_2 *min^{-1} *µmol^{-1}]**					
Fdx7 WT	86.84	51.44		69.14	17.7
Fdx7 S38E	85.87	79.06		82.47	3.41
Fdx7 A92F	67.65	72.06		70.16	2.51
Fdx7 I102E	69.74	60.02		64.88	4.86
Fdx7 W106Y	64.46	47.95		56.2	8.26

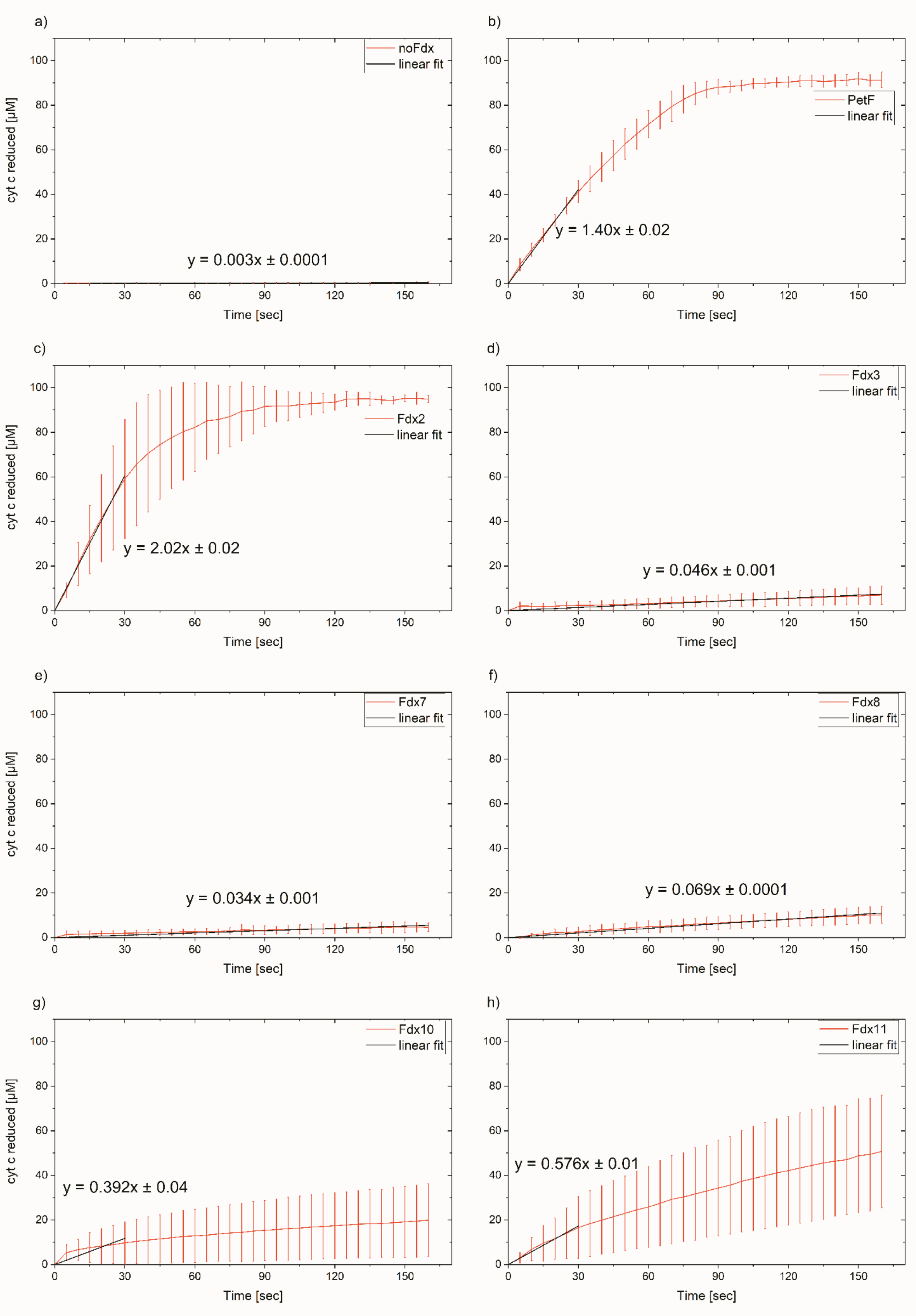

Fig. S7 Fdx:FNR interaction: Time resolved display of results presented in Fig. 3. Electron transfer between FNR and Fdx indirectly measured by NADPH-dependent cytochrome c reduction. 40 nM FNR, 5 µM Fdx and 100 µM cytochrome c were incubated in the presence of 100 µM NADPH. Cyt c reduction was measured photometrically at 550 nm. Error bars depict the mean ± standard deviation for 3-5 measurements from two independent preparations.
